# Paediatric HIV management at primary care level: an evaluation of the integrated management of childhood illness (IMCI) guidelines for HIV

**DOI:** 10.1186/1471-2431-9-59

**Published:** 2009-09-22

**Authors:** Christiane Horwood, Kerry Vermaak, Nigel Rollins, Lyn Haskins, Phumla Nkosi, Shamim Qazi

**Affiliations:** 1 Centre for Rural Health, University of KwaZulu-Natal, Umbilo Road, Durban 4013, South Africa; 2 Department of Paediatrics and Child Health, University of KwaZulu-Natal, Umbilo Road, Durban 4013, South Africa; 3 Department of Child and Adolescent Health and Development, World Health Organization, 20 Avenue Appia 1211, Geneva 27, Switzerland

## Abstract

**Background:**

Integrated Management of Childhood Illness (IMCI) is a WHO/UNICEF strategy to improve child survival in resource poor settings. South Africa adopted IMCI in 1997, and IMCI guidelines were adapted to include identification and management of HIV infected and exposed children. This study describes the validity of the IMCI/HIV algorithm when used by IMCI experts, the use of IMCI/HIV guidelines by IMCI trained health workers in routine clinical practice, and the burden of HIV among children under 5 years attending first level health facilities.

**Methods:**

Seventy seven randomly selected IMCI trained health workers were observed in 74 health facilities in two provinces of South Africa. Consultations were observed with 1357 sick children; each child was reassessed by an IMCI expert to confirm the correct findings. Consent was requested for HIV testing of all children who attended with a parent or legal guardian. Positive rapid HIV tests were confirmed with HIV PCR in children aged less than 18 months. HIV positive children had a CD4 count and HIV clinical staging done.

**Results:**

Of 1064 children with HIV results available, 76 (7.1% CI: 5.7% - 8.9%) children were confirmed HIV positive. IMCI experts using the HIV algorithm classified 54/76 (71.1% CI: 59.5%-80.9%) HIV positive children as suspected symptomatic HIV, and 15/22 remaining HIV positive children were identified as HIV exposed. Therefore, 69/76 (90.8% CI: 81.9-96.2) HIV infected children were identified by IMCI experts. No classification was made for HIV by observed health workers in 899/1357(66.2%) children.

906/1243(72.9%) mothers had been tested previously for HIV, of whom 221(24.4%) reported testing positive. Of 221 children therefore identified as HIV exposed, only 78(35.3%) had been tested for HIV within routine services.

**Conclusion:**

The HIV algorithm is a valid tool for identifying HIV infected and exposed children when correctly and comprehensively implemented. However, it is not being used by IMCI trained health workers in routine practise, leading to a failure to implement life saving interventions.

## Background

South Africa is among those countries worst affected by the HIV epidemic. In 2006, HIV sero-prevalence among pregnant women attending government antenatal clinics was 29.1% [1], and paediatric HIV disease has led to a reversal of the gains that had been achieved in reducing child mortality [2]. Despite the introduction of a programme for prevention of mother to child transmission of HIV (PMTCT) in South Africa, vertical transmission rates of 20.8% have been reported [3], and inadequate testing and follow-up of HIV exposed children is found in PMTCT programmes across the region [4,5]. Mortality among HIV infected children is high; over 50% of untreated African children die in the first 2 years of life [6]. Identification of HIV infected children and early initiation of antiretroviral treatment (ART) would substantially improve mortality [7], but despite availability of free ART, only a minority of children needing treatment receive it [8]. New guidelines from the World Health Organization (WHO) recommend that where virological testing is unavailable, ART should be initiated in children based on clinical diagnosis alone, and the HIV status confirmed as soon as possible [9]. It is, therefore, urgent that strategies are implemented to improve follow-up of HIV exposed children, increase early identification of children with symptomatic HIV, and improve access to ART for children.

**Figure 1:**
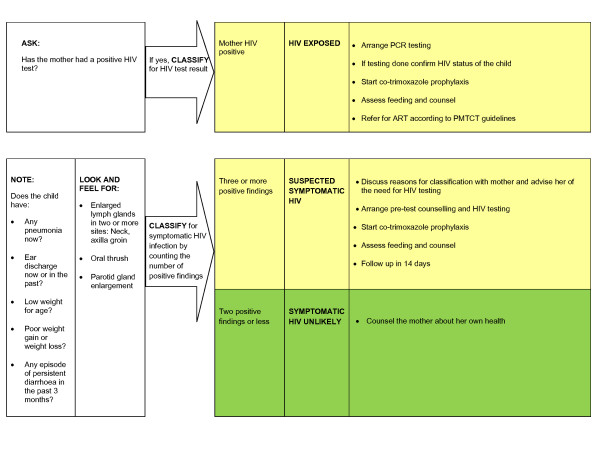
**Current version of the HIV Algorithm, last updated in 2003**.

Integrated management of childhood illness (IMCI) is a WHO/UNICEF strategy to improve child survival in resource poor settings [10], and South Africa is one of 43 African countries to adopt IMCI as the standard of care for children at primary level [11]. IMCI guidelines were adapted to include a validated HIV component to identify and manage HIV infected and exposed children. According to these guidelines, every mother bringing a sick child to a health facility is asked whether she has had an HIV test, and if she reports having tested HIV positive, the child is identified as *HIV exposed*. All children should also be routinely checked for common signs and symptoms found to be most predictive of HIV infection [12]. If three signs are present, the child is classified as *suspected symptomatic HIV *and the carer advised that the child should have an HIV test. These two components make up the HIV algorithm (figure 1), and IMCI includes guidelines for management of HIV infected and exposed children. The HIV/IMCI algorithm has been shown to be a valid tool for identification of children with symptomatic HIV infection [12], and may have a role in identifying those HIV exposed children most at risk of early death [13].

First level health workers are trained to use the IMCI/HIV guidelines during the 11-day IMCI case management course [14], a structured training course supported by comprehensive training materials developed by WHO/UNICEF, and adapted for use in South Africa. Training materials relating to the HIV component were added, and participants learn the practical skills required during clinical sessions, but the overall duration of the training course was not increased. The IMCI/HIV algorithm provides a clear guideline for health workers to identify children for whom HIV testing is indicated, and its effective implementation is an important entry point to care at primary level for HIV infected and exposed children and their families in South Africa, and other high prevalence countries [15].

Previous reviews of IMCI implementation have shown that IMCI training improves health worker performance [16], but implementation of the HIV guidelines has not been evaluated previously. We conducted this study to determine how the IMCI/HIV guidelines are used by IMCI trained health workers, the validity of the HIV algorithm when used by expert IMCI practitioners and in routine clinical practice, as well as the burden of HIV disease among children under 5 years attending first level health facilities.

## Methods

### Study site and population

IMCI trained health workers working in first level health facilities in Limpopo and in Kwazulu-Natal (KZN) provinces, South Africa, were included in this study. IMCI training has been ongoing in both provinces since 1998, and at the time of the study 1325 health workers had been trained in Limpopo Province and 1300 in KZN. Limpopo province has a population of 5.5 million, and is predominantly rural, with high rates of poverty and poor access to basic services[17]. KZN has lower rates of poverty and several large urban centres, but almost 50% of the population still live in rural areas. With a population of 10 million, KZN is the most populous of South Africa's provinces [18]. Antenatal HIV sero-prevalence in 2006 was 39.1% and 21.5% in KZN and Limpopo respectively [1].

In order to evaluate routine IMCI implementation, all IMCI trained health workers currently working in a primary health care (PHC) clinics were eligible to participate. PHC clinics provide first level care to communities and this care is provided mainly by nurses. The district hospital provides support and referral care to PHC clinics in the surrounding area. Study participants were selected by a simple random sample, using computer generated random numbers http://www.random.org, from a list of all IMCI trained health workers working in PHC clinics in each of the two provinces. All observed health workers had attended an 11-day IMCI training course, which included the HIV component. All first level health workers were informed by the Department of Health that a survey of child health practices was to be undertaken, but observed health workers were not told beforehand that they had been selected, or that IMCI in particular was being evaluated.

### Training and Data Collection

Data collection teams consisting of two expert IMCI practitioners and two study counsellors, trained in HIV counselling, were identified for each province. All IMCI experts were experienced IMCI facilitators. Data collection teams were trained by the investigators (CH, KV) for two weeks; this included a refresher of the IMCI/HIV guidelines (theory and clinical sessions) and training on HIV transmission, HIV testing, WHO clinical staging [19], and data collection, including storage and handling of blood samples. A pilot study was conducted in two health facilities, and data collection tools were adapted accordingly.

**Figure 2:**
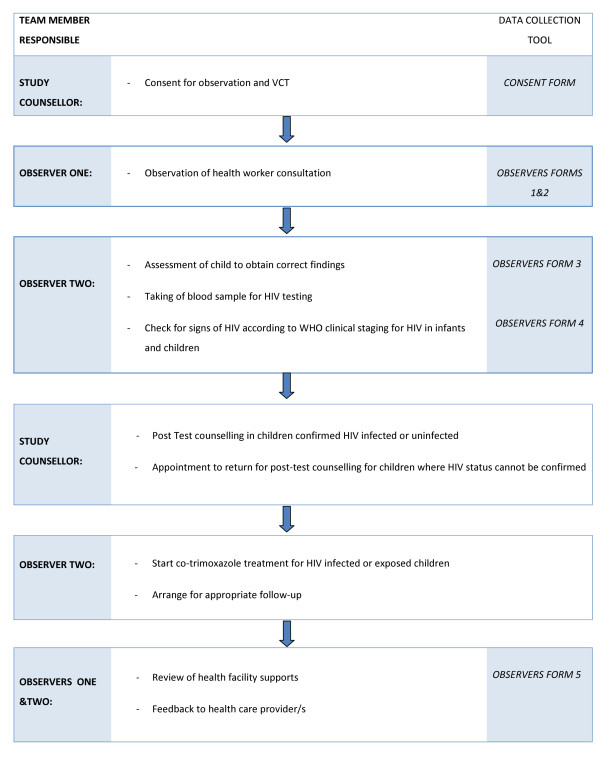
**Flow of study participants through data collection process**.

All sick children aged from 2 months up to 5 years attending the health facility were eligible to participate; consent was requested from carers by study counsellors (figure 2). The consultation by the health worker was observed by an IMCI expert, who recorded the activities and findings without intervening. The same observer was used for all observations of a particular health worker. Thereafter, a second IMCI expert assessed the same child separately and recorded the findings, which were considered the gold standard for analysis of health worker performance. The second IMCI expert was blinded to the findings of the observed health worker. If an IMCI expert was known to the health worker then, if possible, that expert undertook re-assessments of observed children, and did not observe the health worker. No identifying information was recorded about observed health workers or PHC clinics visited. If the management of the child was incorrect, this was changed by the second IMCI expert to ensure all children received appropriate treatment. Each health worker was observed for up to 20 consultations with sick children presenting consecutively to the health facility, or for three days, whichever was shorter.

### Data collection tools

Standardized data collection instruments were used to record the data. Interviews were conducted with health workers to find out about their training in child health, including IMCI. Resources available at the clinic were observed and recorded using a checklist; these included essential medicines and equipment, HIV counselling and testing supplies and services, numbers of children routinely tested for HIV, and whether privacy was available for consultations with children.

Observations were conducted using a standardized checklist based on the IMCI consultation; activities undertaken during the consultation were recorded, including whether relevant history and examination was undertaken, and whether appropriate counselling messages were given to the mother. On completion of the consultation, the observer reviewed the child's card and recorded all IMCI classifications made by the health worker. At no time did the observer interfere in the consultation; feedback was given to the health worker only when all observed consultations were complete. The findings of the IMCI expert who re-assessed the child were recorded on a standard IMCI recording form, which is widely used during training to record IMCI findings.

Teams were visited regularly by the principal investigator, and all completed questionnaires were checked to monitor quality during data collection.

### Consent and ethical approval

Written informed consent was obtained from the carer by the study counsellor in the local language. Consent was requested for observation of the consultation, a second assessment of the child by an IMCI expert, and for HIV counselling and testing of the child. If the carer was not the legal guardian, consent was requested for observation only. If consent was obtained for HIV testing, pre- and post-test counselling was performed by study counsellors. If the child was known to be HIV infected, and this could be confirmed from the medical record, this was recorded without repeating the HIV test.

This study was conducted in partnership with the South African Department of Health and health workers selected were required to participate.

Each child was assigned a study number at enrolment; no names were recorded in the database, and results of HIV tests were linked to clinical data only after completion of the study. Ethical approval was obtained from the Biomedical Research Ethics Committees of the University of KwaZulu-Natal Medical School, Durban, and WHO, Geneva.

### HIV testing

Children over 18 months of age were screened for HIV antibodies using the rapid test Determine™ HIV-1/2 (Abbott, Weisbaden, Germany). All positives were confirmed by a second HIV rapid test using the Smart Check™ HIV1/2 (World Diagnostics Inc, Miami, USA). In children aged below 18 months, if the screening HIV rapid test was positive, 50 μl of whole blood was collected by heel prick using a lancet, and dropped onto a filter paper for the dried blood spot (DBS). The filter paper was transported to Inkosi Albert Luthuli Central Hospital (IALCH) in Durban for PCR testing. The dried blood spots were tested for HIV-1 DNA using the HIV-1 DNA test, version 1.5 (Roche, Branchburg, USA). All children confirmed as HIV infected had 1-2 ml of blood taken in an EDTA tube (Vacuette, GreinerBio-one, Kremsumunster, Austria) which was sent to IALCH for CD-4 cell count; this was performed using the Panleucogated CD4 Epics^® ^XL™ (Beckmancoulter, Galway, Ireland). All blood samples were discarded when HIV testing was complete.

Children under 18 months who could not have their HIV status confirmed immediately had post-test counselling and CD4 testing done by study staff at a follow-up visit. All HIV positive children were started on co-trimoxazole prophylaxis, and assessed for initiation of ART.

### Sample Size

The estimated sensitivity of the IMCI/HIV algorithm when used by IMCI trained health workers compared to IMCI experts was used to calculate the sample size. Based on assumptions that 80% of observed children would be correctly classified for HIV with 10% precision at 95% confidence levels, it was calculated that 62 children classified as *suspected symptomatic HIV *by the expert IMCI practitioners should be sampled.

Health workers were observed for up to 20 consultations to increase the likelihood that every health worker would see at least one case of *suspected symptomatic HIV*. This was based on 2005 antenatal HIV sero-prevalence of 19.3% in the lower prevalence setting of Limpopo [20], and the assumption that one third of children born to HIV infected women become infected, so community prevalence was estimated to be 6.5% for the purpose of sample size calculation.

As the study progressed, it was evident that many health workers were not using the IMCI/HIV guidelines, and an interim analysis was conducted. The sensitivity of the health worker using the HIV algorithm, compared to the IMCI expert was only 26% at the mid-point of the study. Furthermore, the community prevalence in Limpopo was only 2.0%. The sample size was, therefore, recalculated and an additional 15 IMCI trained health workers randomly selected in KZN.

### Data management and analysis

Pre-coded data were double entered, cleaned and validated using Epi-info (version 6.04). Analysis was conducted using SPSS (version 13.0) and Stata (version 9.0). To determine the performance of the health workers, assessments by the health worker were compared to those of the IMCI expert, which were considered to be the gold standard for this purpose. To determine the validity of the HIV algorithm compared to the HIV test results, sensitivity, specificity, positive and negative predictive values, and likelihood ratios were calculated.

## Results

**Figure 3:**
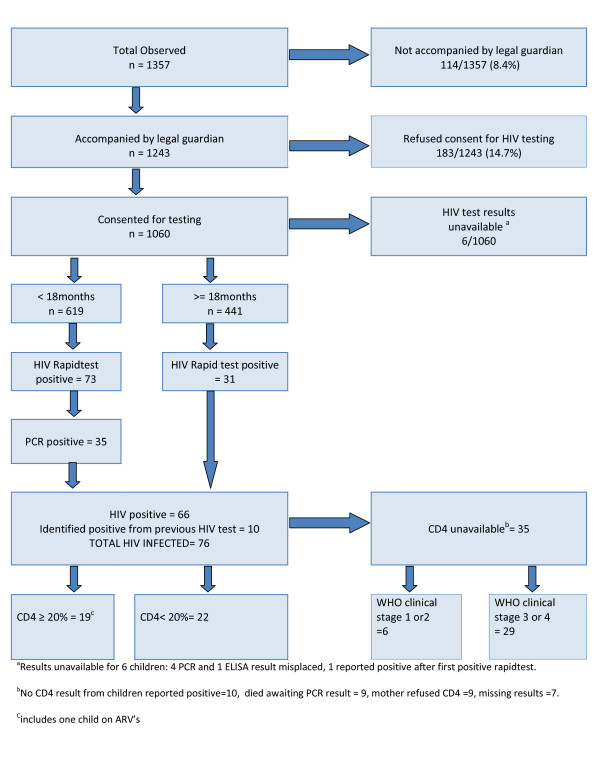
**Outcome of HIV testing for all study participants**.

Consultations of 77 IMCI trained health workers, in 74 primary level health facilities in KZN and Limpopo provinces, were observed between May 2006 and January 2007. Consent was obtained to observe 1357 consultations (figure 3), and each health worker was observed for a mean of 2.7 days (SD 0.68) and 17.7 consultations (SD 4.8). The average age of observed children was 19.6 months (SD 15.0), and 552 (40.7%) were under the age of one year. A total of 499 consultations were observed in Limpopo and 858 in KZN.

### Training and supervision of observed health workers

All observed health workers were nurses, and the average time since IMCI training was 32.2 months (SD 18.4; range 3-88 months). The HIV algorithm was updated in 2003 to its current version (figure1); 19/77 (24.7%) health workers had been trained on a previous version, of whom 8/19 (42.1%) had received specific training on the updated version. All observed health workers were using the current version of the IMCI guidelines.

Of the 77 observed health workers, 55 (71.4%) had no training related to child health, other than IMCI training. One health worker had been trained as an IMCI supervisor and one as an IMCI facilitator; 7 had a diploma in primary health care, which includes a paediatric module; and 4 had attended a short course related to paediatric care, including tuberculosis (1), antiretroviral treatment (1) and immunization (2). Training in HIV/AIDS counselling had been completed by 66/77 (85.7%) health workers.

It is recommended that new IMCI practitioners receive a follow-up visit 4-6 weeks after training to assist in transferring their skills to the workplace; 51 (66.2%) health workers had a follow-up visit by an IMCI supervisor since being trained, of these, 13 (25.5%) had received two visits and 3 (5.9%) three visits. There was no regular, ongoing IMCI supervision.

### Facility supports for IMCI/HIV guidelines

Children were seen in a private area at 50 of 74 (67.6%) health facilities visited. Co-trimoxazole was available in 71 (95.9%) clinics and nevirapine syrup in 58(78.4%). Although 39 (52.7%) clinics reported being able to do HIV PCR tests on children, only 24 (32.4%) had done any PCR tests in the past month, and 32 (41.6%) clinics had not tested any child under 5 years for HIV in the past month.

### HIV results

HIV results were available for 1064 children; 1060 children tested during the study and 10 children reported HIV positive, less 6 children whose results were lost (figure 3). Of these, 76 children were found to be HIV positive, so that HIV prevalence among children attending primary health care clinics was 76/1064 (7.1% CI: 5.7% - 8.9%).

### Mother's reported HIV status

Among 1243 children who attended the clinic with their parent or legal guardian, 906 (72.9%) mothers were reported to have had an HIV test, and 221 (24.4%) were reported as having tested HIV positive. 221 children were, therefore, identified as HIV exposed, of whom 78 (35.3%) had been tested for HIV by routine services.

Among mothers of the 76 HIV positive children, 62 reported having had an HIV test, of whom 43 reported having a positive test, 17 reported testing negative, and 2 did not know the result.

### Indications for anti-retroviral treatment

Of the 76 children confirmed HIV positive, one child was currently on ART. A CD4 count was performed by the study team on 40/75 of the remaining HIV positive children. ART was considered to be indicated in children with a CD4%<20% and those who were WHO stage 3 or 4. When a CD4 result was unavailable, indication for ART was determined by WHO clinical staging alone. ART was indicated in 63/75 (84.0%) HIV infected children (table 1).

**Table 1:** Performance of the HIV algorithm in identifying those children where antiretroviral treatment (ART) is indicated

ART indicated (n = 76)	Number	% (95% CI)	Number Identified by algorithm^a^	Proportion identified by algorithm % (95% CI)
Indicated by CD4<20%	22	8.9 (19.0 - 41.2)	18	81.8 (59.4 - 93.3)

Indicated by WHO clinical stage 3 or 4	60	78.9 (67.4 - 87.2)	50	83.3 (70.0 - 91.5)

Total ART indicated	63	82.9 (71.2 - 90.5)	52	82.5 (69.4-90.8)

ART not indicated by CD4 count or clinical staging	6		1	

ART not indicated by clinical staging, CD4 not available	6		1	

Already on ART	1		0	

Total	76		54	71.1 (59.5 - 80.4)

^a ^classified as suspected symptomatic HIV

### Performance of the HIV algorithm used by the IMCI expert compared to HIV test

Of 1064 children with an HIV test result available, 171 (16.1%) children were classified as *suspected symptomatic HIV *by the IMCI expert; 54 (71.1%) of whom were HIV positive (table 2). Of 22 HIV infected children with insufficient signs for a classification of *suspected symptomatic HIV*, 15 were identified as *HIV exposed*. So, when the HIV algorithm was fully implemented according to the existing IMCI guideline (figure 2), 69/76 (90.8%) HIV infected children were identified as either *suspected symptomatic HIV *or *HIV exposed *(table 2). Of 63 HIV infected children where ART was considered to be indicated, 52 (82.5%) were classified as *suspected symptomatic HIV *(table 1).

**Table 2:** Performance of the HIV algorithm compared to HIV test results

n = 1064^a^	Classified as suspected symptomatic HIV	Identified as HIV exposed	Any HIV classification (either HIV exposed OR suspected symptomatic HIV)
#	171	201	313

# confirmed HIV positive	54	43	69

Sensitivity (95% CI)	71.1% (59.5% - 80.9%)	56.6% (44.7% - 67.9%)	90.8% (81.9% - 96.2%)

Specificity (95% CI)	88.1% (85.9% - 90.1%)	84.0% (81.6% - 86.2%)	75.3% (72.5% - 77.9%)

PPV (95% CI)	31.6% (24.7% - 39.15)	21.4% (15.9% - 27.7%)	22.0% (17.6% - 27.1%)

NPV (95% CI)	97.5% (96.3% - 98.4%)	96.2% (94.7% - 97.4%)	99.1% (98.1% - 99.6%)

Likelihood ratio + (95% CI)	5.99 (4.79 - 7.48)	3.54 (2.77 - 4.51)	3.67 (3.22 - 4.18)

^a ^comprises all children where an HIV test result is known

### Use of the HIV guidelines by IMCI trained health workers compared to IMCI expert

Many health workers (31/77; 40.3%) did not classify for HIV in any child, and 9 did not classify any child correctly for HIV. Although 3/77 health workers did classify for HIV in every child, no health worker classified every child correctly for HIV.

Health workers made a classification for symptomatic HIV in 428/1357 (31.5%) children; of these 342 (25.2%) were classified correctly as compared to the IMCI expert. Among all observed children, 202 (14.9%) were classified as *suspected symptomatic HIV *by the IMCI expert; of these 37 (18.3%) were correctly classified by the health worker.

Health workers classified 84 children as *suspected symptomatic HIV*, including 28/76 (36.8% CI: 24.7% - 51.0%) HIV infected children. Health workers correctly identified 161/223 (72.6% CI: 65.3% - 79.3%) children classified as *HIV exposed *by the IMCI expert. It was not possible to determine the sensitivity and specificity of the HIV algorithm when used by the health worker compared to the HIV test, because 68.5% children were not classified for HIV.

### Health workers management of suspected symptomatic HIV

Of 84 children classified as *suspected symptomatic HIV *by the health worker, 54(64.3%) carers were advised of the need for HIV testing, co-trimoxazole prophylaxis was prescribed for 26 (31.0%) children, and feeding advice was given to carers of 36 (43.0%) children.

## Discussion

This study shows that when the HIV algorithm is applied to all children by skilled IMCI practitioners it is an effective tool to identify HIV infected children, and its implementation could improve access to life saving treatments for HIV infected and exposed children. However, the HIV component of IMCI is frequently not implemented by IMCI trained health workers in routine clinical practice; few children classified as *suspected symptomatic HIV *by IMCI experts were correctly classified by health workers, and most children were not classified for HIV at all. Even when health workers made the classification of *suspected symptomatic HIV*, children were not tested for HIV and essential treatments were not initiated.

There are several possible reasons for poor implementation of HIV guidelines in our setting, IMCI training may not give health workers adequate skills, and some health workers were trained using a previous version of the HIV algorithm. HIV related stigma may make health workers reluctant to mention HIV during a consultation with a child [21], although the high uptake of HIV testing among our study participants suggests that most mothers want information about their child's HIV status. The low positive predictive value (PPV) of the HIV algorithm may also negatively affect implementation. It is important that health workers understand that the HIV algorithm is a screening tool, not a diagnostic test, and that most children identified by the HIV algorithm will test HIV negative. This should be clearly explained during IMCI training, and health workers trained in the appropriate counselling messages to give to a mother when advising her to take her child for HIV testing. If health workers do not understand that such children will frequently test HIV negative and expect this outcome, they may lose confidence in the algorithm and be reluctant to use it. Similarly, if mothers who are advised to take their child for HIV testing do not get clear and appropriate messages, it may make routine checks for HIV in children unacceptable to communities.

Poor implementation of the HIV component may also be an aspect of poor implementation of IMCI overall. Although IMCI implementation has been shown to improve health worker performance [16,22], poor adherence to the guideline has been previously reported [23,24]. Reasons suggested for this include heavy workloads [25], additional time needed for an IMCI consultation [26], and lack of clinical supervision and support. Supportive supervision of IMCI trained health workers has been found to contribute to improvements in correct treatment and counselling [23], but our findings show that many IMCI trained health workers had not received a supervisory visit, and there was no ongoing IMCI supervision.

Poor implementation of the PMTCT program is also highlighted; despite many mothers reporting a positive HIV test, most HIV exposed children had not been tested, and many clinics were not testing any children under 5 years. Strengthening of PMTCT and its linkage with IMCI is required, together with improved access to HIV PCR testing for HIV exposed children. Comprehensive PMTCT implementation and early confirmation of HIV status of all exposed children may reduce the need for an algorithm to identify symptomatic HIV, but it will still have a role for children whose mothers do not disclose their status, and those whose mothers become infected during pregnancy and breastfeeding. In settings where virological testing is not readily available, the algorithm may play an important role in clinical decision making [13].

Despite poor implementation, we have shown that when the HIV algorithm is comprehensively applied to all children by skilled IMCI practitioners, a high proportion of HIV infected children are identified, confirming the findings of a previous validation study [12]. The HIV algorithm is an effective screening tool, and performs better in children where ART is indicated. However, although the sensitivity of the algorithm is above 90%, even in this high prevalence population the PPV is low, and would be even lower in a low HIV prevalence setting. In our population of children almost one third were identified as requiring an HIV test, and it is important to recognise that this represents a significant burden on the health system.

No previous studies have reported HIV prevalence and the burden of HIV disease among children under 5 in this setting; our study shows that undiagnosed HIV infection is common in PHC clinics, and most of these children have advanced disease. Early initiation of ART improves mortality [7], and recommendations now suggest that ART should be initiated in children under 1 year as soon as the HIV status is confirmed [9]. Our findings strongly support the IMCI recommendation to check all children for possible HIV infection.

The strengths of this study include that the IMCI experts were all experienced IMCI facilitators, and able to provide a reliable gold standard. We were able to observe larger numbers of children and health workers than previous health facility surveys [23,27,28], and could, therefore, describe performance with the health worker as the unit of analysis. We were also able to test most enrolled children for HIV, and measure outcomes confidently. Health workers were not given notice of the study team's arrival, a large number of observations over several days reduced subject bias, and data collection tools were designed to minimise interaction between health worker and observer and reduce the chance of performance improving over time. Limitations of the study include: the observer's presence may have influenced health worker performance, some health workers may have 'learned' what was required during the observation period, and some IMCI experts and health workers may have known each other, all of which could have led to bias. In order to avoid interfering during the consultation, we did not evaluate health workers' performance in identifying specific signs, and because of poor implementation we were not able to determine the performance of the HIV algorithm in routine practise. In addition, the sensitivity of HIV rapid tests in children under 18 months has not been fully evaluated, and we were not able to obtain CD4 results for all HIV infected children.

## Conclusion

In conclusion, IMCI has a potential to identify HIV infected and exposed children using the existing guidelines and to provide more and earlier access to care for many children. However, poor IMCI implementation is severely limiting this in routine practice. Further investigation is required to determine the reasons for poor health worker performance, and to provide evidence-based interventions to address poor IMCI implementation. Possible interventions could include strengthening IMCI training, IMCI updates to help maintain skills, as well as increased support and supervision. There is a high burden of HIV disease in PHC clinics and it is critical that these children access treatment if child mortality is to be reduced in South Africa.

## Competing interests

The authors declare that they have no competing interests.

## Authors' contributions

CH was the principal investigator for the study, designed the study, supervised data collection, and participated in the analysis of the data and writing of the paper. KV participated in the design of the data collection tools, oversaw data entry and participated in the data analysis and the writing of the paper. PN and LH participated in the data collection, analysis, and the planning and writing of the paper. NR and SQ advised on the design of the study, the analysis of the findings and the writing of the paper. All authors read and approved the final manuscript.

## Pre-publication history

The pre-publication history for this paper can be accessed here:

http://www.biomedcentral.com/1471-2431/9/59/prepub
